# Cyclin D1 expression and cervical metastases in squamous cell carcinoma of the mouth

**DOI:** 10.1016/S1808-8694(15)31128-9

**Published:** 2015-10-20

**Authors:** Gerson Schulz Maahs, Denise Cantarelli Machado, Emilio Antonio Jeckel-Neto, Vinicius Schenk Michaelses

**Affiliations:** 1 Otorhinolaryngologist/Head and Neck Surgeon. M.S. in Medicine - PUCRS. Member of the Otorhinolaryngology/Head and Neck Surgery Department - PUCRS - Porto Alegre; 2 PhD. Coordinator of the Cellular Biology and Respiratory Diseases Laboratory - Biomedical Research Institute. Professor at the School of Medicine - PUCRS; 3 Biologist and Master in Education (PUCRS), PhD in Medicine (Aichi Medical University, Japan), Full Professor at the School of Biosciences; 4 PhD Student in Clinical Medicine and Health Sciences. Pharmaceutic. Biochemist. Otorhinolaryngologist/Head and Neck Surgeon. Porto Alegre RS PUCRS - Hospital São Lucas e Hospital Mãe de Deus - Porto Alegre RS

**Keywords:** cyclin d1, squamous cell carcinoma, mouth cancer metastases immunohistochemistry

## Abstract

**Summary:**

Cervical metastasis represent the most relevant prognostic factor in carcinomas of the mouth. Clinical and histological factors are associated with the development of cervical metastasis; however, research on molecular factors has been broadly carried out in recent years.

**Aim:**

The aim of this study is to analyze the association of the Cyclin D1 as a risk factor for the presence of cervical metastasis.

**Materials and Methods:**

Cyclin D1 expression was measured and the association between such substance and metastasis was found in 45 patients with mouth cancer treated by the author of this paper. Cyclin D1 presence was checked through the stereological method. Clinical and histological characteristics have been analyzed and associated with metastasis.

**Results:**

Cyclin D1 expression was found in 15 patients but it was not associated with clinical and histological factors related to the presence of metastasis. Such conclusion indicates that Cyclin D1 is an independent protein. The most important predictive aspects related to metastasis development have been clinical staging and vascular embolization.

**Conslusions:**

Cyclin D1, although independent, is not associated with cervical metastasis, while staging and vascular embolization are.

## INTRODUCTION

Epidermoid carcinoma corresponds to more than 90% of malignant mouth tumors, and its survival is modified according to disease staging. When the disease is local, survival reaches 79%, loco-regional diseases yields 42%; and 19% is the survival rate when there are distant metastasis^1^. Clinical, histological, and most recently biological or molecular characteristics are considered prognostic factors, being predictive of cure and survival. The most important prognostic factor in mouth cancer is the presence of neck metastasis, and this makes the diagnosis of distant metastasis fundamental for the oncology treatment planning^2^,^3^.

Cervical metastasis identification is carried out through physical exam and complementary tests, which fail in the diagnosis of 20 to 35% of the clinically negative patients^2^,^3^. Such false negative rate is deemed high and justifies preventive neck treatment. When the primary tumor is treated exclusively, and there is hidden neck metastasis, there is a relevant reduction in patient survival. Patient selection for neck clearance is fundamentally based on the tumor clinical characteristics, as to its size and location^3^. Histopathological characteristics, such as cellular differentiation level and tumor thickness also help in this selection; however, still keeps high failure rates. Tumor biological aspects are being researched in an attempt to identify which patients have a higher chance of developing neck metastasis^3^,^4^.

D1 cyclin is a protein coded by gene CCND1 located in chromosome 11q13. This protein acts on the cellular cycle, accelerating phase G1 and was described as an oncogene in 1991 by Motokura5, who observed that an increase in cyclin deregulates the cell cycle and contributes to tumor genesis. D1 cyclin presence is shown by immunohistochemistry in head and neck cancer, and the higher its expression, the lower patient survival is, thus being considered a prognostic factor by many researchers in carcinomas of the esophagus, breast, uterine cervix, colon, rectum and melanomas^5^,^6^.

In the present investigation, we assessed D1 cyclin expression in a series of 45 patients with mouth epidermoid carcinoma, and its association with neck metastasis. The goal of our study was to try and relate D1 cyclin expression as a risk factor for neck metastasis.

## PATIENTS AND METHODS

### 1. Patients

We studied 45 patients with mouth epidermoid carcinomas who were operated by the author during the period of January 1991 and December of 2001. The patients suffered tumor surgical resection in its place of origin and neck lymphnode clearance. The neck lymphnodes surgery included patients who did not present clinical metastasis (N-), and those from patients who did present clinically diagnosed metastasis (N+).

D1 cyclin expression was carried out in histologic slides obtained from the primary tumor. We reviewed patients' charts and collected pre-surgical clinical data, pathologic exam data from the surgical specimen and post-operative development.

Patients who presented one or more of the following situations were taken off the study:

+ Surgery without curative intention.
+ Other concomitant neoplasias or history of prior tumors.
+ Neo-adjuvant therapy (chemo and radiotherapy).
+ Patients whose chart data were incomplete.
+ Impossibility of defining clinical staging

### 2. Immunohistochemistry

We then obtained the paraffin blocks that had the primary tumors from the patients we were studying. From the main block of each tumor we obtained 4mm thick cross sections.

The sections were placed on 10% poly-L-lysine (Sigma, USA) pre-treated slides and taken to the oven at 60oC for 24 hours.

Paraffin was taken off by means of 10 minute xylene incubation for three times, followed by rehydration in ethanol sequence in decreasing concentrations, starting by absolute ethanol, 90%, 80% and 70% for 3 minutes in each dilution. Following that, the sections were washed three times in distilled water.

Antigenic sites recovery with trypsin digestion was carried out after heating at 37oC in a 0.1% trypsin solution with 0.1% calcium chloride in 7.8 pH. The slides were then incubated in this solution for 30 minutes at 37oC, and were then washed in distilled water.

High temperature antigen exposure was carried out by incubation in glass cradle, with a buffer containing 1mM of EDTA arranged in a 45o angle, in mean power for five minutes and fifty seconds, followed by eight minutes in low power (Panasonic, 1.500W microwave oven).

After recovering the antigenic sites we proceeded blocking endogenous peroxidase with H2O2 at 3%. After a 10 minute incubation, the slides were washed three times with distilled water and once with PBS pH 7.2. The sections were incubated for one hour with primary antigen anti-D1 cyclin (Dako, USA) at the concentration of 1:20, followed by two washings in PBS. After incubation with the primary antibody, the sections were incubated with the biotin-streptavidine system (Dako LSABâ2 System, USA) which consists of an anti-mouse/anti-rabbit antibody incubation biotinilated for 10 minutes, followed by a PBS washing and later incubation with streptavidine conjugated with peroxidase. After 10 minutes of incubation, the slides were washed in PBS and a solution with diaminobenzidine (Dakoâ Liquid DAB Substrate Chromogen System, USA). After washing in distilled water, the slides were counter-dyed with Harris hematoxylin for 15 seconds, washed in three water baths and dipped in an ammonia 37 mM solution. After counter-dye, the slides were washed in three baths of warm water, followed by dehydration in growing ethanol concentrations (80%, 90% and pure, respectively) for three minutes in each dilution. After two treatments for five minutes in xylene, the sections were covered by thin slides assembled in Entellanâ (Merck, Germany).

The slides were analyzed under light microscopy aiming at assessing D1 cyclin expression.

D1 cyclin expression measure was carried out by means of a method called stereologic, which aim is to determine quantitative parameters from bi-dimensional sections of three-dimensional microscopic anatomical structures, by means of geometry and statistics^7^,^8^.

D1 cyclin expression was measured by the count of dyed nuclei divided by the area in mm2 and the value indicated by Qa 9.

**Figure 1:**
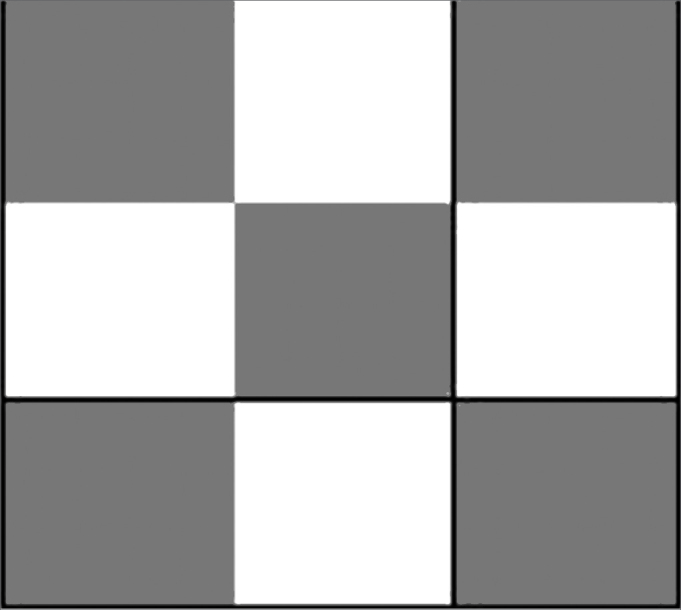
Diagram showing the fields observed in the slide - * The dark area indicated where D1 cyclin labeled nuclei were counted.

Slide analysis was carried out under Olympus AX-70 (Japan) light microscopy, coupled to a video camera. The images obtained were transferred to a computer and counts made with the help of the Image Pro Plus version 4.1 software (Media Cybernetics, USA). On an image taken from a 20x lens we applied a grit that divided it in nine equal fields, according to Figure 1.

From each slide we obtained five values and calculated the average of five Qa obtained.

### 3. Statistical analysis

The quantitative variables were described by average and standard deviation, and percentages were used for the qualitative variables. The group's quantitative variables were compared by means of the Student t test, and for the qualitative variables we used the chi-squared test. Association measures such as the odds ratio and the relative risk with its respective confidence intervals were completely performed. In order to adjust the association between cyclin and metastasis in front of so diverse potential risk factors, we used the multivariate logistical regression.

Survival curves were calculated using the Kaplan-Meyer's method. Death that did not occur because of the primary tumor or from its metastasis were not considered treatment failures, but rather censored observations. The differences between the survival curves were compared by the log rank test. In order to simultaneously adjust the effects of many factors considered and their relation with death, we used Cox regression. The P values below 0.05 were considered statistically significant, and the multivariable models considered P values below 0.1.

For the statistical analysis, data was processed and analyzed with the help of the SPSS (Statistical Package for Social Science) software for Windows and Excel 97, version 11.1.

### 4. Ethics

The study is in compliance with items III.3.i and III.3.t from the Research Regulatory Guidelines and Standards involving Human Beings (Resolution CNS 196/96), as well as Guideline 12 from the International Ethics Guidelines for Biomedical Research Involving Human Beings (CIOMS 1993).

The principles present in the Helsinki Declaration were abided (WORLD MEDICAL ASSOCIATION, 1996), keep confidential the patients' identities, whose data were identified only by the record number in the project.

The project was submitted and approved by the Research Ethics Committee of the Pontifícia Universidade Católica do Rio Grande do Sul (Protocol number 298/01 - CEP).

## RESULTS

D1 cyclin clinical and histologic characteristics of the 45 patients studied may be seen on Table 1.

**Table 1:** Comparison between metastatic and non-metastatic groups as to the following variables: demographics, habits and clinical traits, histology and tumor molecular characteristics.

Variable	With metastasis (n=23)	Without metastasis (n=22)	OR	IC95%	P
**Age, years**	59,0±11,4	57,0±11,0	1,02	0,96 a 1,07	0,543
**Gender, # (%)**					
Males	22 (95,7)	18 (81,8)	4,9	0,5 a 47,7	0,187
female	1 (4,3)	4 (18,2)	-		
**Smoking, n^o^ (%)**					
yes	18 (78,3)	18 (81,8)	0,8	0,2 a 3,5	0,999
no	5 (21,7)	4 (18,2)	-		
**Alcohol intake, #**^o^ (%)					
yes	13 (56,5)	12 (54,5)	0,9	0,3 a 3,0	0,999
no	10 (43,5)	10 (45,5)	-		
**Tongue, # (%)**					
Involved	12 (52,2)	14 (63,6)	0,6	0,2 a 2,1	0,634
Not involved(a)	11 (47,8)	8 (36,4)	-		
**Classification T (tumor), n^o^ (%)**					
T1 and T2(a)	12 (52,2)	16 (72,7)	-		
T3 and T4	11 (47,8)	6 (27,3)	2,4	0,7 a 8,5	0,265
**Clinical staging, # (%)**					
I e II(a)	4 (17,4)	13 (59,1)	-		
III e IV	19 (82,6)	9 (40,9)	6,8	1,7 a 27,1	0,010
**Degree of differentiation, # (%)**					
G1	0 (0,0)	1 (4,5)	0,2(b)	(0,0 a 16,7)	0,462
G2	16 (69,6)	16 (72,7)	0,7	(0,2 a 3,3)	0,878
G3(a)	7 (30,4)	5 (22,7)	-		
**Embolization, # (%)**					
Present	14 (60,9)	4 (18,2)	7,0	1,8 a 27,5	0,009
Absent(a)	9 (39,1)	18 (81,8)	-		
**Nerve involvement, n^o^ (%)**					
Present	14 (60,9)	11 (50,0)	1,6	0,5 a 5,1	0,665
Absent(a)	9 (39,1)	11 (50,0)	-		
**Cyclin, n^o^ (%)**					
[+]	9 (39,1)	6 (27,3)	1,7	0,5 a 6,0	0,598
[-](a)	14 (60,9)	16 (72,7)	-		

Data presented as average ± standard deviation and frequency (percentage). OR: odds ratio; CI95%: Confidence Interval of 95%. (a) Reference category in the calculation of odds ratio; (b) Odds ratio adjusted by Agresti's method.

**Figure 2:**
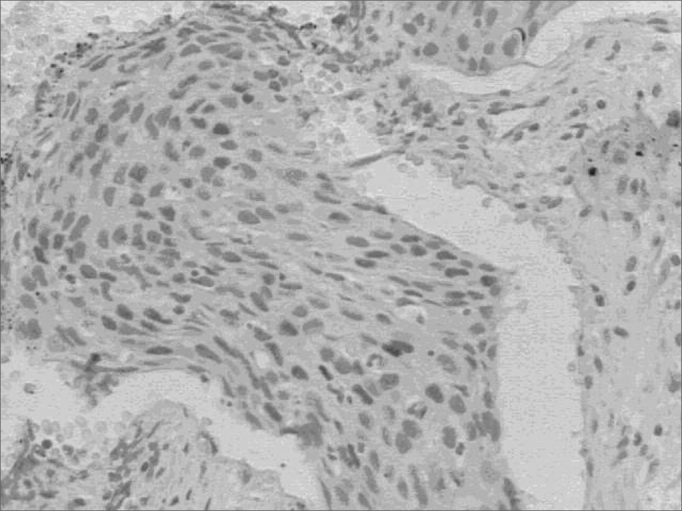
D1 cyclin expression - D1 cyclin expression dyed in Brown in the nucleus of neoplastic cells.

D1 cyclin was positive in 15 (33.3%) cases and negative in the remainder 30 (66.7%). The average of labeled nuclei (Qa) varied between 0.15 and 1.24 in the positive cases. The protein presence in the cell nucleus may be seen in Figure 2.

The protein expression in the cell cytoplasm was also observed in three patients.

The associations and correlations between patients with metastasis (pN+) and those without it (pN-) were carried out base don clinical and histological traits, habits, demographics and D1 cyclin, are depicted on Table 1. The multivariate analysis used to asses the risk of D1 cyclin association with other potential risk factors was carried out and is depicted on Table 2.

**Table 2:** Multivariable logistic regression model which adjusts the cyclin effect [+] when facing many potential risk factors for its association with metastasis.

Variable	OR	CI90%	P
Cyclin [+]	2.7	0.6 a 11.6	0.267
Clinical staging III and IV	16.4	2.8 a 95.5	0.009
Tongue involvement	0.2	0.1 a 0.9	0.068
Vascular embolization	4.3	1.1 a 17.5	0.084
Cellular differentiation G3	8.1	1.2 a 53.4	0.069
Nerve involvement	0.5	0.1 a 2.1	0.460

OR: odds ratio; CI90%: Confidence Interval of 90%.

**Figure 3:**
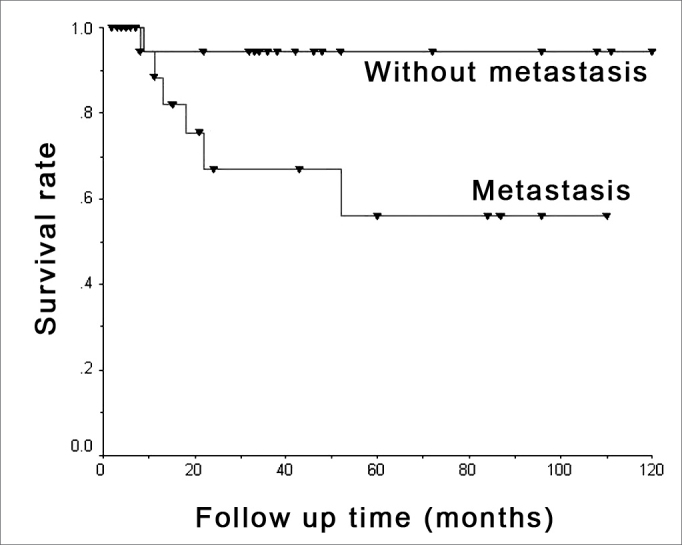
Metastasis and survival - Kaplan-Meier's chart showing death occurrence stratified by metastasis presence or absence.

**Figure 4:**
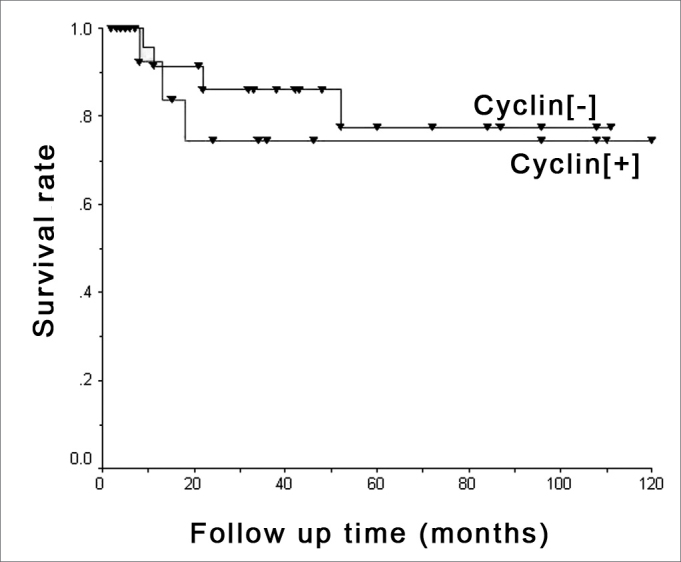
D1 cyclin and survival - Kaplan-Meier's chart showing death occurrence stratified by positive ([+]) and negative ([-]) cyclin groups.

Survival was assessed based on D1 cyclin expression and the presence of metastasis in months, by means of a survival curve of which outcome considered was death due to mouth cancer, which occurred in seven situations. Results may be seen in Figures 3 and 4.

**Figure 5:**
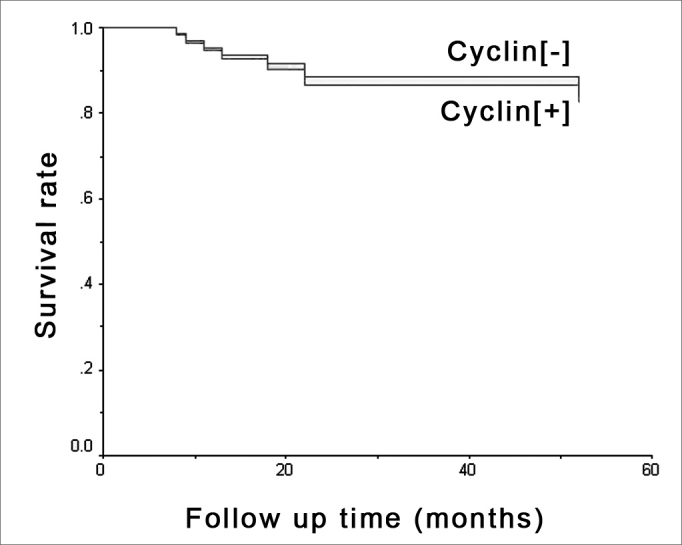
D1 cyclin and survival - Multivaried analysis - Survival chart comparing positive ([+]) and negative ([-]) cyclin groups with adjusted effects in multivariable Cox's regression model, bearing clinical staging, tongue involvement, cell differentiation, vascular embolization and nerve invasion factors.

D1 cyclin effect was adjusted based on potential risk factors for survival assessment and is depicted on Table 3. D1 cyclin effect on survival adjusted by the multivariable Cox regression model containing clinical staging, tongue involvement, cell differentiation, vascular embolization and nerve involvement were studied and are depicted on Figure 5.

**Table 3:** Multivariable Cox regression model which adjusts the cyclin effect [+] when facing many potential risk factors for its association with death.

Variable	RR	CI90%	P
Cyclin [+]	0.8	0.2 to 3.5	0.846
Clinical staging III and IV	4.9	0.6 to 37.9	0.198
Tongue involvement	1.0	0.2 to 5.7	0.964
Vascular embolization	1.6	0.3 to 7.2	0.625
G3 Cell differentiation G3	3.6	0.6 to 20.9	0.226
Nerve involvement	0.4	0.1 to 2.4	0.409

OR: odds ratio; CI90%: Confidence Interval of 90%.

## DISCUSSION

D1 cyclin was expressed in 15 (33.3%) patients from the sample, which is in agreement with published results that vary between 30 and 40%^10^, ^11^, ^12^, although by a distinct methodology of immunohistochemistry reading. The literature on D1 cyclin and cancer assesses the presence of cyclin by counting the nuclei labeled by a group of neoplastic cells, which usually vary between 500 and 1000 cells, in percentages. By means of such method, the investigator needs to count a total of neoplastic cells, regardless whether they are marked or not, and after that, recounting the cells that express the protein, and calculate the percentage. Besides being a labor-intensive method, it bears a higher risk of distortions in counting. The choice of cutting point that separates the positive from the negative D1 cyclin expression is arbitrary. Han et al.^13^, consider it positive when there is more than 5% of labeled nuclei. Ravi et al.^14^, Itami et al.^11^ and

Capaccio et al.^12^, consider the D1 cyclin expression only significant when present in over 10% of the tumoral cells. El-Naggar et al.^15^ consider values above 50% as an overexpression of this protein. Mineta et al.16 consider values between 0-50% being of low expression, and those above 50% as being of high expression. Schoelch et al.10 use the negative score when there is no expression, + for up to 33% expression, ++ between 33 and 66% and +++ above 66% of protein expression in tumor cells.

The stereological method used in this investigation assess the protein expression in the nuclei by tumor area, is more reliable^8^,^9^. The protein expression in the cell cytoplasm was also observed as described in other studies^17^.

D1 cyclin may be expressed in different intensity levels. The term “high expression” is defined by the labeling of more than 50% of the neoplastic cells^10^. In the present study, Qa results obtained for the 15 positive patients varied from 0.15 to 1.27, and the differences of the values found were not significant. It was not possible to establish the cyclin expression in low and high expression because of patient sample size; however, the quantitative analysis of Qa values was carried out.

D1 cyclin expression in the 15 positive patients was not constant in the whole tumor surface area. In all the cases, protein expression analysis was carried out in the region of greatest labeling in the slide, showing tumor areas that did not express the protein. This lack of labeling constancy may be justified by the fact that about 20% of the neoplastic cells are under mitosis^18^. Considering this asymmetrical expression distribution, one could question the studies in which the researchers used biopsy specimens, as reported by Michalides et al.^19^, where D1 cyclin expression false negatives were observed in biopsy material when compared to the surgical specimen; nonetheless, further studies are required in order to measure which is the true sensibility of D1 cyclin expression in biopsy material compared to a surgical specimen. This is relevant, since false negatives occurring in the biopsy material would compromise the clinical application of such marker when a treatment decision is to be made, for example, whether or not to indicate neck clearance in a patient clinically classified as N0.

As to gender, smoking and alcohol intake, there was no relation with the presence of neck metastasis and the invasion of lymphnode capsule, which confirms that they do not represent risk factor for neck metastasis^16^.

Age was also not related to the presence of neck metastasis and lymphnode capsule invasion, although very young and very old patients develop tumors with more aggressive behavior^20^.

As to location, there was also no association with the presence of metastasis. The 14 T1 and T2 patients with tongue epidermoid carcinomas presented metastasis in six cases, while the remaining 14 T1 and T2 patients classified as having cancer in other parts of the mouth presented metastasis in six cases. Location is not important when the tumor is large, as is the case with T3 and T4 tumors that already bear high metastasis development risk individually.

Tumor location in the mouth was associated to lymphnode capsule invasion. The tumors that involved more than one location in the mouth had greater involvement of the lymphnode capsule; with a p value of 0.049.

Twelve of the 28 T1 and T2 patients presented histologic metastasis (42.8%), a value close to expected, which is of 20 to 40%. As to the 17 T3 and T4 patients, 11 had metastasis (64.7%) in agreement with the literature^21^. There was no statistical relation between tumor size and metastasis (P = 0.265). We also did not find significant association between tumor size and metastatic lymphnode capsule invasion (P = 0.089).

Regarding the classification N, histology showed that of the 25 negative cases, eight (32%) were false negatives and 17 (68%) were true negatives. As to the 20 N+ patients, histology revealed five (25%) false positive patients, and in 15 (75%) neck metastasis was confirmed. The P value found was of 0.001 for neck metastasis and 0.047 for capsule invasion. The number of false positives was of 25%, corresponding to what has been described in the literature, which is 20%, while the rate of false negatives was of 32%, while the value reported in the literature lays between 20 and 35%^22^,^23^. Metastasis patients' stratification was not studied in the present investigation, due to the small number of patients allocated in each subdivision N.

Clinical staging was significant in the association with neck metastasis. When the staging of a mouth cancer patient is defined, it is considered tumor size and the clinical presence of metastasis by neck palpation. Since patients classified as positive N are in stages III and IV, the value found is not surprising and is directly associated with the incidence of false positives and false negatives on neck palpation, which in the present study was of 25% and 32% respectively. Neck palpation and the T classification alone were not significant, and this by itself justifies the use of a staging process.

The degree of cell differentiation was not associated to the presence of neck metastasis or with lymphnode capsule invasion. The presence of only one patient with well differentiated epidermoid cancer made it impossible to observe the relationship between the degree of differentiation and the risk of metastasis development.

Nerve sheath invasion was not associated to the presence of neck metastasis, nor to the invasion of lymphnode capsules. Nerve sheath invasion happened in 14 (60.9%) of the 23 metastatic patients; however without statistical meaning. There was also no association between nerve sheath invasion and lymphnode capsule invasion.

Vascular embolization was predictive as far as metastasis presence is concerned. Woolgar^24^ found 78% of vascular invasion in N positive patients and 70% nerve sheath invasion in another sample also of 45 patients with mouth epidermoid carcinoma. In the present study we found 60.9% for both situations; however with different meaning when we observe the results of the proper statistical significance tests, and this shows the relevance of data analysis tests, thus avoiding misinterpretations.

D1 cyclin expression is not associated to metastasis. D1 cyclin quantified by Qa value also did not change the results. The lack of studies published in the literature using the stereological method makes it impossible to compare quantitative results. Cyclin was also not associated to metastatic lymphnode capsule invasion.

In the literature, the association between D1 cyclin expression and the presence of neck metastasis is ambiguous. The results of the present investigation agree with those presented by Han et al.^13^, Michalides et al.^19^,^25^ and Bellacosa et al.^26^. However, the papers from Frachiolla^27^, Itami11, Masuda^28^, Tapia et al.^29^, Capaccio et al.^30^ and Mineta et al.16 showed that D1 cyclin is indeed associated with the presence of neck metastasis. Results are contradictory and the reasons may be the following: D1 cyclin expression differ in the different organs of the body; the very condition of the surgical specimen such block tumor and formaldehyde time until it is fixed; cutting point used to define what is protein overexpression and a standard reading methodology avoiding interpretation subjectiveness.

D1 cyclin multivaried analysis with the most relevant prognostic factors did not change the results, showing that D1 cyclin expression does not depend on the other factors studied, agreeing with the findings from Capaccio et al.^30^ and Mineta et al.^16^.

The survival curve of the 45 patients in the sample shows that D1 cyclin positive patients have a lower tendency to survive when compared to negative patients, although the statistical significance was weak.

Survival analysis concerning metastasis reveals that neck metastasis patients present lower survival rates, and confirms the meaning of neck metastasis as a prognostic factor associated with mouth cancer.^3^,^31^, ^32^, ^33^.

Multivaried analysis shows that D cyclin and neck metastasis are independent factors as to their relation with survival, agreeing with the results from Han et al.^13^. These authors did not find associations between D1 cyclin and the presence of metastasis in gastric cancer, and found an association with patient survival. Michalides et al.^19^,^25^ studied head and neck carcinomas and Bellacosa et al.^26^ studied patients with larynx cancer and also did not report correspondence between metastasis and D1 cyclin expression; nonetheless, they did find relations with patient survival.

In the present investigation, neck metastasis was significant in survival reduction, while D1 cyclin did not show the same statistical significance, only showing a trend that patients with higher cyclin expression had worse survival rates.

## CONCLUSIONS

The present investigation leads us to conclude that:

a. D1 cyclin expression seems to be independent from mouth epidermoid carcinoma.
b. It is very likely that D1 cyclin is not associated with the presence of neck metastasis.
c. Vascular embolization and clinical staging were the most significant factors as neck metastasis predictors in the cases hereby studied.
d. Neck metastasis was significant in the survival of our patients in this study, while the same can not be said about D1 cyclin.
